# Experiences of online group support for engaging and supporting participants in the National Health Service Digital Diabetes Prevention Programme: A qualitative interview study

**DOI:** 10.1177/13558196231212846

**Published:** 2023-12-14

**Authors:** Wang Chun Cheung, Lisa M Miles, Rhiannon E Hawkes, David P French

**Affiliations:** 1 Research Assistant, School of Biological Sciences, Faculty of Biology, Medicine and Health, 5292 University of Manchester, Manchester, UK; 2 Research Associate, Manchester Centre for Health Psychology, Division of Psychology and Mental Health, Faculty of Biology, Medicine and Health, 5292 University of Manchester, Manchester, UK; 3 Research Associate, Manchester Centre for Health Psychology, Division of Psychology and Mental Health, Faculty of Biology, Medicine and Health, 5292 University of Manchester, Manchester, UK; 4 Professor of Health Psychology, Manchester Centre for Health Psychology, Division of Psychology and Mental Health, Faculty of Biology, Medicine and Health, 5292 University of Manchester, Manchester, UK

**Keywords:** diabetes prevention, digital interventions, group support

## Abstract

**Objectives:**

The National Health Service Digital Diabetes Prevention Programme is a nine-month behavioural intervention for adults in England at risk of type 2 diabetes. This qualitative study aimed to explore how service users engaged with the group support available within the programme.

**Methods:**

The majority of participants (*n* = 33), all service users, were interviewed twice via telephone, at 2–4 months into the programme, and at the end of the programme at 8–10 months. Semi-structured interviews covered participants’ experiences of online group support functions and how such groups served as a route of support to aid participants’ behavioural changes. Data were analysed using manifest thematic analysis.

**Results:**

The majority of participants valued the format of closed group chats, which provided an interactive platform to offer and receive support during their behaviour change journey. However, engagement with group chats reduced over time, and some participants did not find them useful when there was a lack of common interests within the group. Health coaches helped to promote engagement and build rapport among participants within the group chats. Participants reported mixed experiences of discussion forums.

**Conclusions:**

Programme developers should consider how to optimise online group support to help service users make behavioural changes, in terms of format, participant composition and use of health coach moderators. Further research is required to better understand who might benefit most from ‘group chat’ or ‘discussion forum’ support. Health coach moderation of online support groups is likely to facilitate engagement.

## Introduction

Type 2 diabetes mellitus (T2DM) is a significant health care problem in the United Kingdom (UK), with more than three million people diagnosed in England.^1^ It is estimated that T2DM and its associated complications currently cost the National Health Service (NHS) £10 billion each year^1,2^; thus, its prevention is a public health priority.

Randomised controlled trials of diabetes prevention programmes implemented globally^3,4^ have found that progression to T2DM can be slowed or prevented through providing behavioural interventions that target weight reduction through improved diet and increased physical activity. These programmes were initially developed as face-to-face group sessions. Following this international evidence, the NHS Diabetes Prevention Programme (NHS-DPP) was launched in England in 2016. By 2020, over 200,000 people had attended the first session, and over 100,000 people had attended at least 60% of the programme’s sessions.^5^ Initial results have been promising, suggesting a reduced population incidence of T2DM in England.^5^ Nevertheless, face-to-face group-based formats are not suitable for everyone. Barriers to participation include logistical challenges such as travel, childcare and work commitments.^6,7^

Digital interventions have the potential to offer behaviour change support at scale.^8^ A meta-analysis demonstrated the preliminary effectiveness of digital interventions through reductions in bodyweight in people at high risk of T2DM.^9^ With the aim to expand the reach of the NHS-DPP, a pilot study of a digital version of the programme was conducted. Initial results have shown clinically significant reductions in bodyweight and HbA1c levels at 12 months.^6^ NHS England subsequently launched the NHS Digital Diabetes Prevention Programme (NHS-DDPP) in 2019. Upon referral by general practitioner or self-referral after assessment by an online diabetes risk tool,^10^ service users were directed to one of four digital service providers that were commissioned to deliver the service in their local geographical area.

The four NHS-DDPP providers delivered their own versions of the digital programme, based on key intervention components detailed in the NHS England service specification.^11^ The providers utilised a variety of online features, including, but not limited to, smartphone apps (e.g. for tracking weight, physical activity and diet), e-learning materials (e.g. articles and videos) and health coach support (e.g. via telephone/video calls and messaging). Three of the providers also included online group support in their digital programmes. Table 1 summarises the differences in group support features offered across these three providers.

**Table 1.** Characteristics of online group support across the three NHS-DDPP providers.^a^

Phase of the programme^b^	Provider 1	Provider 2	Provider 4
Core (weeks 1–12)	Health coach moderates group chats of approx. 10–15 people (format similar to WhatsApp)	Discussion forum among all service users on the NHS-DDPP (format similar to Facebook)	Discussion forums among all service users on different programmes offered by the provider, moderated by health coaches, comprising different groups that users can join depending on their sociodemographic characteristics or interests (format similar to Facebook)
	Leader board (interactive group function to record steps of group participants)		
Maintenance (weeks 13–40)	Discussion forum among all service users on different programmes offered by the provider (format similar to Facebook)	Discussion forum (as above)	Discussion forums (as above)
	Leader board (as above)		

Note. Providers are labelled 1, 2, 3 and 4 in this manuscript to preserve anonymity for provider organisations. The labels 1, 2, 3 and 4 do not correspond to labels A, B, C, D used in a previously published article.^12^

^a^At the time of data collection, no support groups were delivered by Provider 3. This provider has since introduced a ‘group pathway’ that includes a group support function.

^b^During the core phase of the programme, providers offered relatively more contact with service users (e.g. weekly). Support gradually tapered off during the maintenance phase.

The literature to date has extensively explored the role of online health communities for people living with long-term health conditions^13^ and the self-management of T2DM specifically.^14,15^ For example, qualitative research evaluating a web-based self-management programme for people living with T2DM found that service users particularly valued the inclusion of an online peer support forum providing them with the opportunity to interact with others on the website, normalise any negative emotions and feel ‘part of a community’.^14^

In the literature on people in diabetes prevention programmes specifically, qualitative research has found that service users participating in face-to-face group support valued sharing their experiences, making social comparisons and having a common purpose and a group ethos.^16–19^ More recently, qualitative research conducted as part of the pilot face-to-face NHS-DPP found that service users described the positive impact of forming lasting social networks that was important for their ongoing participation in the programme.^20^

A recent systematic review has shown that diabetes prevention interventions with a variety of interactive digital features (e.g. educational content, health/outcome tracking and online health coaching) that included social support achieved clinically significant weight loss and improved T2DM-related outcomes.^21^ This review suggested that online support (e.g. via other participants) and face-to-face or phone support (e.g. via family, friends and support staff) were equally effective in digital diabetes prevention interventions.^21^ The pilot study of the NHS-DDPP also revealed that service providers that delivered peer support had better T2DM-related outcomes compared to those providers which did not.^6^ This is consistent with a study of a digital T2DM prevention programme reporting that group participation and support were significantly associated with reductions in bodyweight and HbA1c.^22^

Thus, while the literature suggests that group support is an important component of effective digital T2DM prevention programmes, we are yet to understand how service users experience and engage with these group support features. Further, providers of the NHS-DDPP have included group chats, group discussion forums and health coach moderation as key modes of delivery of group support, but the current literature has not differentiated between these forms of support, nor explored how participants engage with these different formats. Specific objectives of this study were to explore:

1. How do participants experience and engage with group support available within the programme?
2. How do health coaches offer support to participants through support groups?
3. How do participants offer each other support through online support groups?

## Methods

Methods are reported in accordance with the consolidated criteria for reporting qualitative research^23^ (see S1 in the online supplement).

### Study design

This research employed a longitudinal design, where qualitative interviews asked service users about their experiences and engagement with the online group support features of the NHS-DDPP. Interviews were conducted firstly at 2–4 months into the programme and then towards the end of the programme at 8–10 months.

### Participants

During the time of this study, service users were given a choice of attending either a digital or a face-to-face service and participants in this study had chosen to take part in the digital programme. Eligible participants were those who were 2 months into the programme at the point that interview invitations were sent out. The participants were all service users of the programmes offered by Providers 1, 2 and 4. This was because Provider 3 did not offer a support group function at the time of the interviews. Each participant is described below by their gender, age and which provider they used.

### Procedures

Service users on the NHS-DDPP were invited to participate in this study between February and April 2021 and interviews took place during this time period. Service providers sent out invitations to people at 2–4 months into the programme and study details were provided in the participant information sheet. A purposive sampling approach was adopted where follow-up recruitment emails from providers targeted specific sociodemographic characteristics to allow a diverse range of participant recruitment. Further details on the sampling and recruitment methods are provided in a recent study.^12^

Interviews were conducted by two members of the research team (LMM and REH, both Research Associates with training in qualitative methods) and lasted between 30 and 60 min. LMM and REH explained to interviewees that the aims of the research were to understand participants’ experiences and engagement with the NHS-DDPP and how participation in the programme helped the interviewees make behavioural changes. The majority of participants were interviewed twice via telephone. Conducting interviews at two time-points allowed: (a) temporal comparison of individual experiences of the programme across 9 months, (b) better reflection of variations in service delivery across providers and (c) the perspectives of participants to be captured both when they were actually using the programme and after they had completed the programme to minimise risk of lack of recall.

Full audio-recorded verbal consent was obtained via telephone prior to commencing the interview, and sociodemographic characteristics (age, gender, ethnicity and postcode) were recorded. Field notes were made following each interview and recruitment was stopped when the researchers (LMM, REH and DPF) judged there to be no new content being discussed in the final two interviews for each provider. Interviews were recorded using an encrypted audio recorder, anonymised and transcribed verbatim.

### Topic guides

Interviews were semi-structured. Both interviews focused on personal experiences and engagement with group online discussion forums, the moderated support delivered by online group chats, health coaching and how online group functions served as a route of support to aid participants’ behavioural changes. Follow-up questions from the second interview were tailored to each participant, according to their individual responses from the first interview. The interviews also discussed participants’ understanding and use of behaviour change techniques delivered in the programme, the results of which have been reported separately.^12^ The topic guides for the two sets of interviews are available as S2 in the online supplement.

### Analysis

As the research team wanted to understand participants’ views and experiences of engaging with the online group support functions, data were analysed using thematic analysis^24^ and coded at the manifest (or descriptive) level, to stay close to the language of the participants. Data were analysed from a realist perspective, viewing the data as representing the truth of participants’ experiences, but where participants’ interpretations were defining their subjective realities. Researchers sought to understand those subjective realities.

NVivo software (version 12) was utilised to facilitate coding of the interview data. Both interviews from each participant were analysed as a single unit (i.e. interviews conducted at two time-points were first combined as a file in NVivo software), and the first interview was coded before the second one. Interviews were inductively coded by one researcher (WCC), with the aim of identifying patterns in the data according to the different participant demographics and the different types of group support delivered across each of the NHS-DDPP providers (e.g. open group discussion forums and closed group chats). WCC and LMM had regular review meetings during the coding process and all authors regularly met to discuss the data, with illustrative extracts and themes refined through discussion. We did not member check these results, as this was a secondary analysis of interview data,^12^ and previous advocates have later acknowledged that this process is philosophically flawed.^25^

### Researcher positioning

The research team members who conducted the interviews have a background in public health (LMM) and health psychology (REH), including previous experience of delivering behaviour change programmes in local communities (REH). This gave the researchers a strong understanding of the type of behaviour change that can be engendered by supporting people who are at risk of developing long-term health conditions.

The wider team (DPF, LMM and REH) has worked on the evaluation of the face-to-face and digital NHS Diabetes Prevention Programmes for the last 5 years at the time this analysis was written, and DPF has worked on other T2DM projects over the past 20 years. The backgrounds of the wider team may have influenced some of the questions asked in the interviews (e.g. with more of a focus on the individuals rather than wider socioeconomic status constraints) and the interpretation of some of the findings in relation to how health coaches offer support on the programme.^12^

The lead author (WCC) has a background in Biomedical Sciences and was not part of the wider study team, thus viewed the data independently from previous work conducted by the team. Therefore, whilst the wider team were able to situate the current study findings with previous work conducted on the NHS-DDPP, WCC provided an independent perspective on service users’ insights into the online group support offered in the programme.

No members of the research team are currently living with T2DM or are known to be at risk of developing T2DM.

We also sought views from our Patient and Public Involvement and Engagement group, a four-person group all of whom were either at risk of developing T2DM or living with T2DM. This group was convened in 2016 for this programme of research and the group reviewed the topic guide prior to data collection. During this feedback, the group suggested re-wording some of the questions and including additional questions (including whether the programme met the expectations of service users). The topic guide was amended in light of the group’s feedback prior to data collection.

## Results

More participants took part in the first interview (*n* = 33) than the second (*n* = 26).

Overall, 55% of participants were female with a median age of 57 years. Forty-eight percent of participants were from areas of lower deprivation (Deciles 1 to 6).^26^ The majority of participants (79%) were White British. See Table 2 for details.

**Table 2.** Demographic data of the interview participants (*n* = 33).

Demographic characteristics			Total
NHS-DDPP provider^a^	Provider 1	12	33
	Provider 2	10	
	Provider 4	11	
Age (years)	Median (range)	57 (21–76)	33
Gender, *n* (%)	Female	18 (55)	33
	Male	15 (45)	
Ethnicity, *n* (%)	White British	26 (79)	33
	Asian	3 (9)	
	White other	2 (6)	
	Black	2 (6)	
Index of multiple deprivation,^a^ *n* (%)	Decile 1 (most deprived)	3 (9)	33
	Decile 2	1 (3)	
	Decile 3	1 (3)	
	Decile 4	2 (6)	
	Decile 5	4 (12)	
	Decile 6	5 (15)	
	Decile 7	2 (6)	
	Decile 8	5 (15)	
	Decile 9	3 (9)	
	Decile 10 (least deprived)	7 (21)	

^a^Index of multiple deprivation scores, ranging from 1 (representing the 10% most deprived areas in England) to 10 (representing the 10% least deprived areas in England).^26^

### Participants’ experiences with the support groups available within the programme

#### Group chats

Across the three providers, only Provider 1 delivered a group chat function (akin to WhatsApp), while the other providers delivered peer support via discussion forums. The majority of participants on Provider 1’s programme had positive feedback on the group chats, particularly during the first 3 months of the programme, when the chats were also moderated by a health coach (known as the ‘core phase’). Participants described giving each other feedback and adapting their own behaviour change plans and goals. This was particularly valued when they shared common interests with other members of the group chat, which participants found ‘reassuring and helpful’ (female, 50 years, Provider 1). As two participants explained:

Really good, because there’s other people in the group as well and with the group chat on there you listen to how they’re doing. They give feedback on what I’m doing, so it’s kind of like a group thing, we’re all keeping each other going. There’s a lady who is called [the health coach] who coordinates everything and she looks after us, she keeps us going. So, yeah, it’s kind of like a mini fit club. (Male, 44 years, Provider 1)

I didn’t think I would engage in that, just purely because it’s not my kind of thing. But I did, and it was very, very helpful because everybody was on the same sort of wavelength. It has spurred you on to get involved, to ask questions. Other people have asked questions that you may have had, so you can see that answer. It’s been very supportive, and also you learn from each other. And the chat has been in an educational format really, and everyone gets involved, what their thoughts are. (Female, 53 years, Provider 1)

However, the dynamic of the group chat depended on the group composition. For example, the majority of participants who chose not to engage with the group chat described the content as often unrelatable to their own experiences and said they did not identify with others in the group. Sociodemographic characteristics, particularly age and gender, influenced participants’ perceptions of how they might benefit from interaction with others:

Most of the other people that are on it are women. There are no other men on it at all, and they’re just talking about knitting and stuff, that’s what they’re talking about and I’m just not interested. (Male, 56 years, Provider 1)

My initial thoughts were, ‘I would be put in a group of similar type of people with similar sort of issues, more locally based.’ But it turned out that it was a whole mix of different people with different abilities and troubles, from quite elderly people down to in their thirties … The chat was around their own personal ailments, which I couldn’t really relate to. I haven’t got arthritis, I haven’t got 14 grandchildren. So, I found that a bit odd, that I was in such a varied group from all around the country. (Male, 57 years, Provider 1)

Despite many participants discussing the supportive nature of these chats, engagement gradually reduced towards the end of the programme. Some participants referred to their busy life outside the programme, while some felt that they did not need as much support once they had developed their own healthy habits to continue on their own:

At the beginning we were all obviously a bit more enthusiastic. I think as time’s gone on that’s got less and less … We’ve still got our own lives to lead. But, then, it could be that people have got more used to the programme and so they haven’t got so many questions to ask. (Female, 64 years, Provider 1)

#### Leader board (interactive group function to record group participants’ step counts monitored by step trackers)

Most male participants valued the competitive element of the leader board function, which was offered by Provider 1 to record cumulative steps of the group. Engaging with the leader board allowed social comparison with other users on the programme and enhanced their sense of achievement and enthusiasm for continuing to make behaviour changes. As one participant explained:

Sometimes you feel a bit competitive on it because obviously it helps you to see how many steps the other people have done and you look at yourself and you’re thinking, ‘I’m fifth out of however many people and the other guys have done more steps.’ It makes you feel that competitiveness to go and do more steps, so it’s helping you stay healthy and I do like a bit of competitiveness. So it is good to see that. (Male, 44 years, Provider 1)

Interestingly, female participants rarely described this type of upward social comparison in such positive terms. Rather, females described this competitive aspect of the leader board as putting stress and pressure on them, in turn negatively affecting their confidence and enthusiasm towards making behavioural changes:

I also found looking at the stats of the cohort and realising that I had five or 10% progress then everybody could see that. I could see that some people were nearly 100% of their progress. And it just felt like competition that I didn’t want to be involved in with people, that I couldn’t sort of catch up to within the cohort. And that felt uncomfortable, with the other people understanding just how little progress I made during the time that I was in the programme. (Female, 50 years, Provider 1)

#### Group discussion forums

Group discussion forums (akin to Facebook) were a feature delivered across Providers 1, 2 and 4. However, such forums received a mix of positive and negative comments. Some said it facilitated idea sharing, which encouraged participants to support their behaviour changes. They said it was motivating knowing that others were in a similar situation they were:

I found it kind of reassuring that other people are having the same struggles and finding their own route through. Some people, I think, found it helpful to just post and not necessarily need any feedback from anybody else, others were asking for help and guidance. [Posts included] menu suggestions, suggestions around things that my coach had given me and new ideas about bringing other food types into menu choices. So, yeah, other people are going on the same journey and kind of thinking wish you well. (Female, 63 years, Provider 2)

One participant disclosed that she was agoraphobic, meaning the face-to-face programme was not an option for her, so the discussion forums were particularly important for her to benefit from interaction with others to ‘get that shared experience that you’re all on a similar journey, similar goals’ (female, 48 years, Provider 4).

A handful of participants described ‘lurking’ on the forums.^27^ They did not contribute personally to the content and discussion but still benefitted through reading and learning from others’ comments without the requirement of open communication. Thus, some did not need to directly engage with the forums to find recognition and socially compare themselves with others, and instead received information from the groups in a passive way. Participants described being reassured just knowing the discussion forum was there if they needed it:

Only read some of their messages or comments. But I’ve not actually engaged with people. It’s not something that perhaps is natural to me, which I think is probably why I went down that route in the first place. But it’s there and I’m aware of it. I’m just nosey. I just like to look what people are saying to be honest. It’s quite useful to know that, you know, there’s quite a lot of people like this. And it’s nothing to be embarrassed [about]. (Male, 48 years, Provider 2)

In fact, others said they opted for the digital service instead of a face-to-face service because they did not want to interact with other people and thus avoided the discussion forums altogether. This finding accords with previous research.^28^ Some participants also attributed this level of disengagement to not being able to relate to the other users posting on the forums, a similar barrier to some participants’ engagement with the group chats offered by Provider 1. Notably, some participants supported the principle of discussion forums, acknowledging their role in facilitating idea sharing and supporting behavioural changes, but chose not to engage as it did not align with participants’ own perceptions of how they preferred to communicate with others:

I just don’t do social media. I’m uncomfortable in these environments. I don’t particularly like them. Therefore, it’s not a natural communication venue for me. And there was a wider group with various other people, which is unassociated – not had any dealings with. (Male, 57 years, Provider 1)

It looked good, but I don’t like groups. I don’t work well with groups. I don’t mind them, but I don’t naturally gravitate towards them, and so I’ve not really seen the sort of desperate need to do it. But I know they’re there, and I suppose if you are someone who would like that, you know, and get a lot from it, then they’re probably really useful. It’s nothing to do with the app or the availability. It’s just not my thing. (Female, 66 years, Provider 2)

### How health coaches offer support to participants through support groups

Health coach moderation of group support was delivered by Provider 1 via group chats and by Provider 4 via open group discussion forums. However, only participants from Provider 1’s programme described the benefit of health coach support via this programme feature. Participants from Provider 4’s programme did not discuss health coach moderation. This may be due to health coaches on this programme offering reactive support rather than proactive support via the group discussion forums.^29^ Overall, these participants valued the health coach moderation within the group chats, describing this as key to providing constructive advice and guidance for supporting their behavioural changes. As one participant said:

The coach feedback was always very positive, or very helpful suggestions, rather than negative. If you were feeling negative, they always turned it into a more positive way to look. That was good. Then we did feedback to each other quite positively in the group. That was very encouraging. (Female, 53 years, Provider 1)

Participants said that, occasionally, interaction in the online group chat was not as active unless the health coach was present to stimulate conversation. Some participants particularly valued the proactive interpersonal skills of the health coach, which facilitated engagement with this programme feature:

It’s really helpful actually, especially when the group’s quiet like that. You just needed one person to try and jolly everybody up really. So, she was incredibly important – because, without her interaction, I wouldn’t have had any interaction with the group at all, there was just nothing there at all. She literally was the last man standing when it came to trying to communicate with the group. So, without her, it would have been absolutely pointless really, so she did great. (Female, 50 years, Provider 1)

The level of social interaction and support within the group greatly decreased when health coaches reduced the frequency of contact after the core phase of the programme (from month three onwards). In some cases, this negatively affected participant engagement:

By that point, I probably disengaged from the programme ... [The health coach] wasn’t sort of bringing a daily group discussion to the table. (Female, 50 years, Provider 1)

### How participants offer each other support through online support groups

Participants who engaged regularly with both the group chats and discussion forums valued the camaraderie built within the group networks, often describing as reassuring with ‘no judgement’ (female, 48 years, Provider 4). This was particularly true for those participants on Provider 1’s programme, which offered closed group chats. As participants felt they were in similar situations with each other, they shared successes and setbacks. They said this enhanced their motivation to continue:

It’s been quite useful because we keep each other track or we discuss the bad days. We say, ‘Don’t worry about that, you know what you’ve got to do to stay on track, so just don’t worry about a bad day.’ (Female, 67 years, Provider 4)

Other service users in the online groups played a critical role in stimulating conversations and idea sharing. The majority that engaged with the group network valued the regular communication, which allowed them to share personal experiences of what worked or did not work for them during their behaviour change journey. They described sharing ideas with other members in their group chat such as recipes, and discussing ways to fit exercise into busy schedules. In turn, this helped participants to reflect upon and adjust their own goals and plans, illustrating that support from other participants is conducive to facilitating behaviour change:

One lady was saying she wasn’t feeling too well so she hadn’t done brilliantly in following the programme. And quite a few of us said to her, ‘I hope you’re feeling better, and just try and get back on track when you can.’ So, we do keep in touch like that. And … [if somebody has] a recipe people take pictures of it when they’ve done it, so we can all see what it looks like. And they say if they liked it or what they would change slightly, if they were going to, so that’s helped. (Female, 64 years, Provider 1)

The recipes, people have asked questions about adapting them. People have suggested things they’ve tried and we’ve all sort of said what we like. What other people have liked that perhaps you’ve overlooked and haven’t tried. Discussions – lots of discussions about fitting in the exercise … Someone was suggesting how they’ve adapted the exercise to their lifestyle, well, actually, yes, that might work better than the way I was doing it. (Female, 53 years, Provider 1)

## Discussion

Overall, there were differences in participants’ experiences and engagement with the online group support functions depending on the format (‘closed’ group chat vs ‘open’ group discussion forums). The majority of participants valued group support in the form of closed chats, which provided an interactive platform to empower each other and share ideas during their behaviour change journey. However, these chats were only perceived as useful if participants shared common interests with others in the group. Health coach moderation of group chats was key to fostering engagement and behavioural changes. Participants had mixed feelings towards the open group discussion forums – some found this support useful for idea sharing, but others found this form of communication uncomfortable. Mutual support from other participants on the same programme could serve as a route for making behavioural changes, but participants had to willingly engage with the group support in order to experience these benefits.

There is a dearth of literature that has specifically investigated the comparative merits of closed group chats versus open group discussion forums, although both are widely used in digital behavioural interventions. The non-standardised way in which studies describe such peer-based social media features is a barrier to this comparison.^30^

This study highlighted the important role that digital group support provided for some participants in facilitating behavioural changes. Participants reported sharing recipes, asking for advice from others and encouraging peers. This type of group support has also been reported as benefits of face-to-face T2DM prevention programmes.^16–19^ However, the usefulness of digital support in this study was only perceived as beneficial if participants could relate to others in the group (e.g. sociodemographic characteristics or similar interests), and the group chats seemed more conducive to offering behavioural support compared to the open group discussion forums. Recent research from a face-to-face diabetes prevention programme suggested that participants benefited not only from receiving but also by *providing* social support,^19^ which might explain why participants from the present study valued the interaction and empowerment facilitated via the group chats. Some participants described difficulties in establishing group cohesion and common interests in the open group discussion forums. This might be due to these larger forums not reaching a ‘critical mass’ of engaged users to deliver sufficient tailored support, as indicated in a previous systematic review.^30^

The opportunity to interact with others sharing a common experience was particularly valued by participants who engaged with these digital groups, which was perceived as reassuring. This concurs with qualitative research on group support in weight-loss groups, which helped establish a shared identity.^31^ These findings also align with the literature exploring the role of online support groups for people living with diabetes.^14,15^ Further, health coach moderation of the group chats seemed vital to both enhance user experiences of the group support functions and to promote continued engagement. When health coach moderation ceased after the first 3 months, engagement in the group chats greatly decreased. The literature has already established the key role that health coaches can play in helping service users to make behavioural changes in digital interventions,^19,32,33^ suggesting a human element may still be required to provide tailored behaviour change support in digital interventions. This includes research on the digital and face-to-face versions of the NHS-DPP, which has found health coaching vital in supporting participants to understand and use behaviour change content in the programme.^12,34^

A recent quantitative analysis exploring engagement with the NHS-DDPP found that the group support functions were very rarely used across the provider programmes, although there was some engagement with the group chats during the first 3 months of the programme when health coach moderation was also provided.^35^ The current qualitative study complements these previous findings. Service users suggested that the health coaches helped to increase engagement and rapport within the group chats, and this format of support received more positive feedback compared to experiences with the group discussion forums which was mixed. This highlights the importance for health coaches to be trained in interpersonal and group management skills in addition to skills supporting individual behavioural changes.^36^ Further, the current study adds context to *how* service users engaged with these groups. Although previous research has found that users rarely posted or interacted with comments on the open group discussion forums,^35^ some service users in this study reported to read group posts without further engagement, describing that it was reassuring to know the groups were there if they needed them. The literature has already distinguished between the ‘posters’ and ‘lurkers’ in online support groups,^27^ suggesting that users can still benefit from online group support passively. Thus, although engagement with the group support appeared low in the NHS-DDPP,^35^ these digital functions could still be providing support, guidance and reassurance to some users who are not actively participating.

Finally, the leader board group function was particularly valued by male participants. This finding is in line with a recent study which found that, compared to females, males preferred competitive tasks.^37^ Previous research exploring group processes in a T2DM prevention programme has suggested that making social comparisons can adversely affect self-efficacy for those who struggled with their own goals and plans.^19^ In the present study, this seemed true for some female participants, who described the leader board as pressurising and decreased their confidence to continue.

## Limitations

There are three main limitations to this study. First, although the age and gender characteristics of our participants were similar to that of the pilot NHS-DDPP, our sample was characterised by lower levels of deprivation and less ethnic diversity compared to that of the pilot programme.^6^ It is likely that our participants will not be representative of all participants involved in the NHS-DDPP or other digital behaviour change programmes.

Second, some of our findings were solely based on the experiences of participants at a single provider. This could potentially limit how applicable these findings are beyond that provider.

Third, at the time the interviews were conducted, Provider 3 did not deliver any group-based online support element. It has since introduced such support.^29^ Further studies could compare and substantiate user experiences of group support delivered by Provider 3.

## Conclusions

This study has highlighted features of group support that have wider implications relevant to digital health interventions beyond the NHS-DDPP, including the preferred format (i.e. a group chat appeared to be preferable to a group discussion forum), composition of groups and important role of health coaches as group moderators. Future research in this area would benefit from clear reporting of these key features of group support, to enhance our understanding further. The authors suggest it is advisable to appreciate the inherent differences of how participants view support through closed group chats and open discussion forums and to differentiate between these different formats.

Further research is warranted to better understand the types of people that might benefit most from closed group chats and whether different sociodemographic groups might benefit from different features of digital group support. It would also be fruitful to assess associations between health outcomes (e.g., bodyweight and HbA1c) and different types of group support to establish whether certain group support features optimise effectiveness in digital behaviour change interventions.

## Supplemental Material

Supplemental Material - Experiences of online group support for engaging and supporting participants in the National Health Service Digital Diabetes Prevention Programme: A qualitative interview study Supplemental Material for Experiences of online group support for engaging and supporting participants in the National Health Service Digital Diabetes Prevention Programme: A qualitative interview study by Wang Chun Cheung, Lisa M Miles, Rhiannon E Hawkes and David P French in Journal of Health Services Research & Policy.
